# Intraperitoneal mitomycin C improves survival compared to cytoreductive surgery alone in an experimental model of high-grade pseudomyxoma peritonei

**DOI:** 10.1007/s10585-019-09991-0

**Published:** 2019-09-20

**Authors:** Olaf Sørensen, Anders Mikal Andersen, Stein Gunnar Larsen, Karl-Erik Giercksky, Kjersti Flatmark

**Affiliations:** 1 Department of Gastroenterological Surgery, Norwegian Radium Hospital, Oslo University Hospital, Montebello, 0310 Oslo, Norway; 2 Department of Pharmacology, Norwegian Radium Hospital, Oslo University Hospital, Montebello, 0310 Oslo, Norway; 3 Department of Tumor Biology, Institute for Cancer Research, Norwegian Radium Hospital, Oslo University Hospital, Montebello, 0310 Oslo, Norway; 4 Norwegian Radium Hospital, Oslo University Hospital, University of Oslo, Montebello, 0310 Oslo, Norway

**Keywords:** Intraperitoneal chemotherapy, Pseudomyxoma peritonei, Mitomycin C, Hyperthermia, Rat model

## Abstract

Pseudomyxoma peritonei (PMP) is a rare cancer commonly originating from appendiceal neoplasms that presents with mucinous tumor spread in the peritoneal cavity. Patients with PMP are treated with curative intent by cytoreductive surgery (CRS) and hyperthermic intraperitoneal chemotherapy (HIPEC). The value of adding HIPEC to CRS has not been proven in randomized trials, and the objective of this study was to investigate the efficacy of intraperitoneal mitomycin C (MMC) and regional hyperthermia as components of this complex treatment. Xenograft tissue established from a patient with histologically high-grade PMP with signet ring cell differentiation was implanted intraperitoneally in 65 athymic nude male rats and the animals were stratified into three treatment groups; the cytoreductive surgery group (CRSG, CRS only), the normothermic group (NG, CRS and intraperitoneal chemotherapy perfusion (IPEC) with MMC at 35 ºC), and the hyperthermic group (HG, CRS and IPEC at 41 ºC). The main endpoints were survival and tumor weight at autopsy. Adequate imitation of the clinical setting and treatment approach was achieved. The median survival was 31 days in the CRSG, 60 days in NG and 67 days in HG. The median tumor weights at autopsy were 34 g in CRSG, 23 g NG and 20 g in HG. In conclusion, the addition of IPEC with MMC after CRS doubled the survival time and reduced tumor growth compared to CRS alone. Adding regional hyperthermia resulted in a modest improvement of treatment outcome.

## Introduction

Pseudomyxoma peritonei (PMP) is rare cancer that most commonly originates from a ruptured mucinous neoplasm in the appendix [1]. It is characterized by accumulation of mucinous tumor in the peritoneal cavity which ultimately leads to abdominal distension, pain and bowel obstruction. Cytoreductive surgery (CRS) aiming at complete removal of all visible cancer tissue and hyperthermic intraperitoneal chemotherapy (HIPEC) have emerged as standard-of-care for treatment of patients with PMP. Observational studies have demonstrated that introduction of CRS and HIPEC in the treatment of PMP has resulted in improved survival compared to repeat debulking surgery, which was the previous standard treatment, with an increase in 10-year overall survival from 20–30% up to 70% [2–5]. However, PMP is a heterogeneous disease, and high-grade histology and incomplete tumor removal are parameters that are associated with inferior survival [6, 7].

The value of adding HIPEC to CRS in PMP has never been assessed in randomized trials and has therefore not been definitely proven in humans. During HIPEC, the peritoneal cavity is continuously perfused with a heated chemotherapy solution, aiming to provide high intraperitoneal drug concentration combined with limited systemic absorption and toxicity [8]. A commonly used chemotherapeutic agent in HIPEC for PMP is mitomycin C (MMC), which has several essential qualities suggesting its suitability for local administration; direct cytotoxic effect, wide anti-tumor spectrum and non-cell cycle specific effect [9]. In vitro experiments suggest enhanced efficacy of MMC in combination with hyperthermia [10, 11], but the benefit of adding hyperthermia to intraperitoneal chemotherapy with MMC is poorly documented and should be further examined.

The purpose of this experimental study was to investigate the role of intraperitoneal chemotherapy and regional hyperthermia as components of CRS–HIPEC. In a rat model of high-grade PMP with signet ring cell differentiation, the impact of intraperitoneal chemotherapy perfusion (IPEC) with MMC was evaluated under normothermic and hyperthermic conditions and survival and tumor growth was assessed.

## Animals and methods

### Animals

Sixty-five locally bred Rowett nude male rats with median weight 209 g (160–258) were used. The animals were maintained under specific pathogen-free conditions and food and water were supplied ad libitum. Housing and all procedures involving the animals were performed in accordance with protocols approved by the Animal Care and Use Committee (Approval ID #3158). Three groups of rats were defined by treatment regimens combining the anti-tumor treatment modalities CRS, IPEC and hyperthermia: The cytoreductive surgery group (CRSG), CRS without IPEC; the normothermic group (NG), CRS and IPEC at 35 ºC which is equal to the body core temperature of the rats; the hyperthermic group (HG), CRS and IPEC at 41 ºC which is a temperature commonly used in HIPEC in patients.

### Tumor

The patient-derived xenograft model was originally classified as peritoneal mucinous carcinomatosis (PMCA) according to Ronnett's classification [12]. The PMCA-3 orthotopic tumor model was previously characterized with respect to growth pattern in mice, microscopic features and immunohistochemical profile [13]. By the Peritoneal Surface Oncology Group International classification [1], the PMCA-3 model is a high-grade PMP with signet ring cell differentiation. Mucinous tumor tissue was harvested from the peritoneal cavity of locally bred female BALB/c (nu/nu) mice and 500 µl was immediately injected into the peritoneal cavity of 4–5 week-old rats. The treatment procedures of CRS and IPEC were performed after median 21 days (19–24).

### Cytoreductive surgery

All treatment procedures were conducted under sterile conditions. General anesthesia was initialized by isoflurane and maintained by subcutaneous injections of a mixture of tiletamine, zolazepam, xylazine and butorphanol [14]. A subcutaneous injection of dalteparine 70 IU/kg (Fragmin, Pfizer, Limoges, France) was administered immediately before the treatment procedure. A midline incision allowed examination of the peritoneal cavity and assessment of tumor distribution. The macroscopic appearance of the tumor model was similar to that in patients suffering from PMP with free mucin and solid lesions of soft consistency, most frequently located on the larger omentum, splenic surface and splenic hilum, liver surface and liver hilum, gonadal fat pads and parietal peritoneum. Peritoneal cancer index (PCI) was determined in seven locations by lesion size: PCI = 0, no tumor; PCI = 1, tumor < 2 mm; PCI = 3, tumor > 2 mm. Overall PCI was calculated as the sum of PCI in the individual locations as outlined in Table 1 with a maximum score of 21, modified from calculation of PCI in animal experiments previously described in [15, 16]. Free mucin was collected using a syringe. During CRS, a bipolar coagulation device was used for resection of peritoneal tumor and organs. The aim of CRS was complete removal of all tumors on the peritoneal surface, but diffuse tumor distribution, including in liver hilum, prevented complete resection of tumor lesions. Intestinal resections were not performed. Residual tumor lesions with a diameter up to 2.5 mm were accepted, which is in line with what is accepted in the treatment of patients with PMP [7]. The weight of mucin and solid lesions removed at surgery was registered. After CRS, the peritoneal cavity was rinsed with saline 0.9% and completeness of cytoreduction (CC) score was determined according to the maximum size of residual tumor: CC = 0, no tumor; CC = 1, tumor < 2.5 mm; CC = 2, tumor > 2.5 mm [6]. Thereafter, the abdominal wall of the animals in the CRSG was closed with two layers with continuous polyfilament suture 3 − 0. The animals in the NG and the HG were prepared for IPEC and placed in a custom-made rack with the abdominal wall elevated to imitate the coliseum position [17–19].

**Table 1:** Comparison of treatment groups at the time of cytoreductive surgery and intraperitoneal chemotherapy

	Cytoreductive surgery group n = 20	Normothermic group n = 20	Hyperthermic group n = 20	*p*-value
Body weight (g)	204 (160–239)	214 (178–249)	208 (175–258)	0.29^a^
Tumor distribution (assessed according to the peritoneal cancer index)				
Greater omentum	3 (3–3)	3 (2–3)	3 (2–3)	
Liver hilum and surface	2 (1–3)	3 (1–3)	2 (1–3)	
Perisplenic	1 (0–3)	2 (1–3)	2 (1–2)	
Mesentery	1 (1–3)	1 (1–3)	1 (1–3)	
Gonadal fat pads	3 (1–3)	3 (2–3)	3 (2–3)	
Diaphragm	0 (0–0)	0 (0–0)	0 (0–0)	
Parietal peritoneum	1 (0–2)	0 (0–3)	0 (0–3)	
Overall peritoneal cancer index	11 (9–15)	13 (8–16)	11 (8–15)	0.22^a^
Solid tumor weight (g)	1.0 (0.5–3.0)	1.3 (0.5–3.5)	1.4 (0.7–2.8)	0.15^a^
Mucin weight (g)	1.4 (0.3–3.4)	1.5 (0.5–4.1)	1.8 (0.3–2.9)	0.23^a^
Splenectomy performed (# animals)	8	13	11	0.16^b^

Median values are shown (min–max)

^a^Kruskal–Wallis test

^b^Chi-square test, linear-by-linear association

### Intraperitoneal chemotherapy

The peritoneal perfusion system consisted of silicon tubes, an intravenous infusion tube and a container holding the peritoneal perfusion fluid (PPF). A peristaltic pump provided a perfusion flow rate of 40 ml/min and heating of the PPF was achieved by inserting the inflow tube into a temperature regulated water bath, as previously described [17]. Peritoneal perfusion was initiated with 150 ml saline 0.9%. When the desired temperature was reached, a single dose of MMC 1.5 mg (Medac, Hamburg, Germany) was added, whereupon IPEC was conducted for 90 min with an open peritoneal cavity. The mean inflow and outflow temperatures were registered at 10-min intervals by a dual input digital thermometer (TMD90, Wavetek Meterman, Long Branch, New Jersey, USA) calibrated at The National Institute of Technology (Oslo, Norway). In parallel, the rectal temperature was registered using a digital patient thermometer. The MMC concentration in the PPF was monitored by collecting 20-µl samples at 10, 30, 60 and 90 min. Samples were diluted 1:10 and stored at -70 ºC until analysis by high performance liquid chromatography [14]. In both groups the positions of the inflow and outflow catheters were reversed after 45 min, primarily in order to reduce the risk of thermal injury to the tissue exposed to the slightly higher temperature of the PPF at the inflow site in the HG but also to provide a more uniform intraperitoneal distribution of MMC. In the HG, ice and water were used for external cooling of the animals during IPEC to prevent body core overheating. After IPEC, the peritoneal cavity was rinsed with 250 ml saline 0.9% at temperature 35 ºC, whereupon the abdominal wall was closed as described above.

Five animals died during or immediately after treatment; three animals (one in each treatment group) died of bleeding. Two additional animals died in the HG, probably because of overheating. Survival analyses are presented for the animals that completed the treatment procedures (20 animals in each treatment group); including all animals based on intention-to-treat did not influence conclusions. All surgical procedures and administration of IPEC were conducted by one investigator who has extensive experience in clinical CRS and HIPEC (O.S.).

### Follow-up

For postoperative analgesia, carprofen (Rimadyl^®^, Orion Pharma, Animal Health, Oslo, Norway) 5 mg/kg was administered subcutaneously once daily on days 0–3. Body weight was registered daily during the first week. Experienced animal research technicians that were blinded for treatment group allocation monitored animal welfare and decided when the individual rats were to be terminated. Humane end points were abdominal distention as a sign of tumor and ascites, signs of pain, inactivity, malnutrition and jaundice. Animals were sacrificed under general anesthesia (isoflurane) by intracardial injection of pentobarbital. Rats with no sign of intraabdominal tumor were terminated on day 110. Autopsy was performed in all animals and the weight of ascites and solid tumor was registered.

### Statistics

Statistical analyses were performed using the Statistical Package for the Social Sciences^®^ program, version 18.0 (SPSS GmBH, Chicago, Illinois, USA). Comparisons of non-categorical parameters [overall PCI, body weight, relative weight loss, amount of solid tumor, mucin and ascites, and area under the time-concentration curve (AUC)] were conducted by two-sided Mann–Whitney test with exact significance and Kruskal–Wallis test with asymptotic significance as appropriate. Comparison of one categorical parameter (splenectomy yes/no) was conducted by Chi-square test, linear-by-linear association. Survival curves were calculated from the date of CRS and IPEC using the Kaplan–Meier product-limit method and differences between groups were analyzed using the log rank test. P values < 0.05 were considered significant.

## Results

### Tumor distribution at surgery and treatment characteristics

There was no significant difference between the treatment groups with respect to tumor load or distribution at the time of treatment. Median overall PCI was 11 (CRSG), 13 (NG) and 11 (HG). The median weight of resected solid tumor was 1.0 g (CRSG), 1.3 g (NG) and 1.4 g (HG) and the median weight of mucin removed was 1.4 g (CRSG), 1.5 g (NG) and 1.8 g (HG). Assessment of residual tumor after CRS showed a score of CC-1 in all animals except for one animal in the CRSG in which tumor on the small bowel allowed only CC-2. The characteristics of the animals, details of tumor distribution and the weight of tumor and mucin at the time of surgery are summarized in Table 1.

**Fig. 1:**
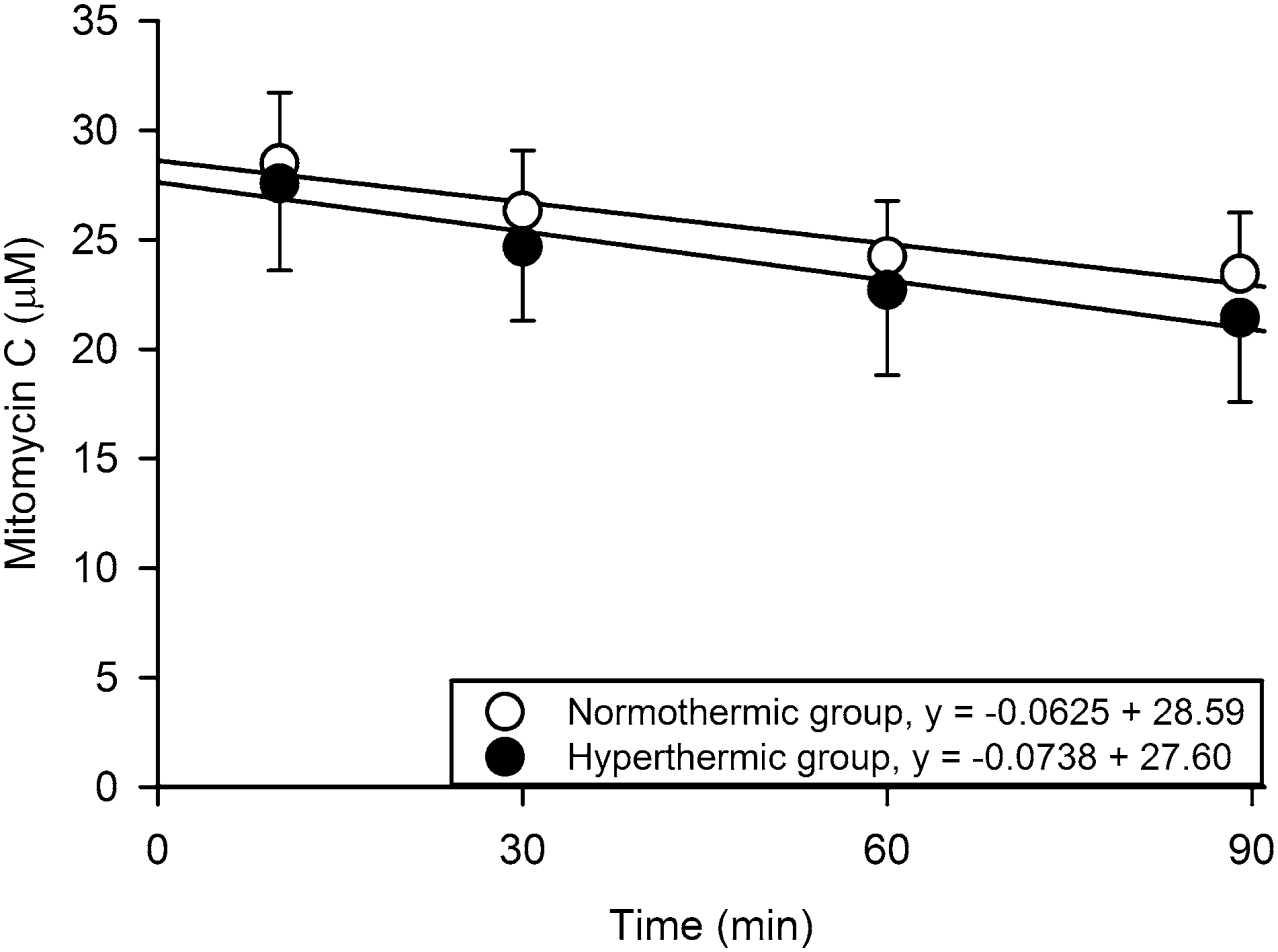
Time–concentration curves of mitomycin C (MMC) in the peritoneal perfusion fluid during intraperitoneal chemotherapy perfusion over 90 min in the normothermic group (35 ºC) and the hyperthermic group (41 ºC). Trend lines are described by equation and accuracy

**Fig. 2:**
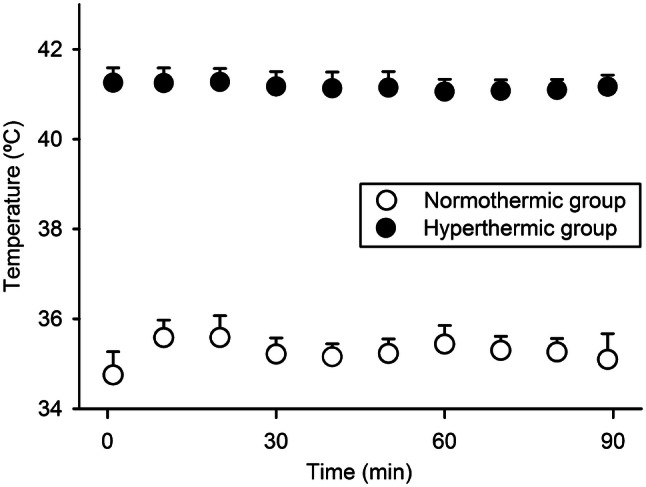
Temperature of the peritoneal perfusion fluid measured during intraperitoneal chemotherapy over 90 min in the normothermic group (35 ºC) and the hyperthermic group (41 ºC)

**Fig. 3:**
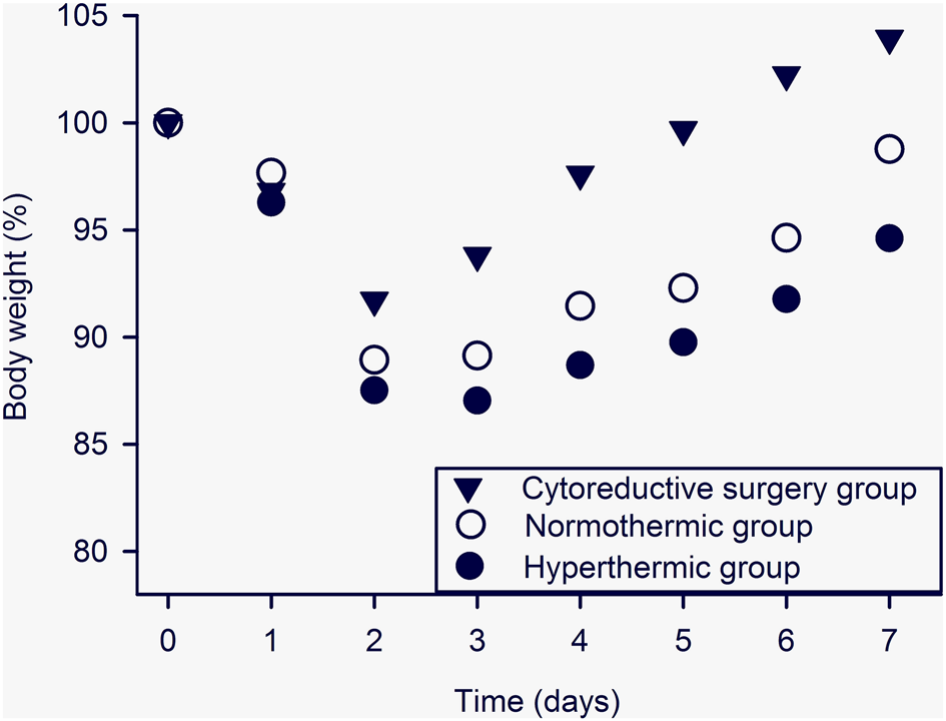
Relative weight loss in the cytoreductive surgery group, the normothermic group and the hyperthermic group over 7 days after treatment. Results are expressed by the mean of 20 animals in each group

The MMC concentration in the PPF was in both groups acceptably stable during the course of IPEC and decreased from 28.6 to 23.0 µM in the NG g and from 27.6 to 21.0 µM in the HG (Fig. 1). The intraperitoneal bioavailability of MMC, expressed by mean and standard deviation (SD) of AUC (µM min) in the PPF, was 2306 (220) in the NG and 2153 (297) in the HG (p = 0.07). The measured temperatures of PPF were in line with the intended level which defined the IPEC groups. Mean (SD) temperature was of 35.3 ºC (0.2) in the NG and of 41.2 ºC (0.1) in the HG (Fig. 2). The core temperature increased moderately during the procedure, in the NG from 32.7 ºC (0.6) to 34.6 ºC (0.7) and in the HG from 34.3 ºC (1.5) to 37.6 ºC (1.2). Maximum relative weight loss occurred on days 2–3 and was 8% in the CRSG, 11% in the NG and 13% in the HG (Fig. 3) (CRSG vs. NG, p = 0.01; CRSG vs. HG, p < 0.001; NG vs. HG, p = 0.13).

### Survival

**Fig. 4:**
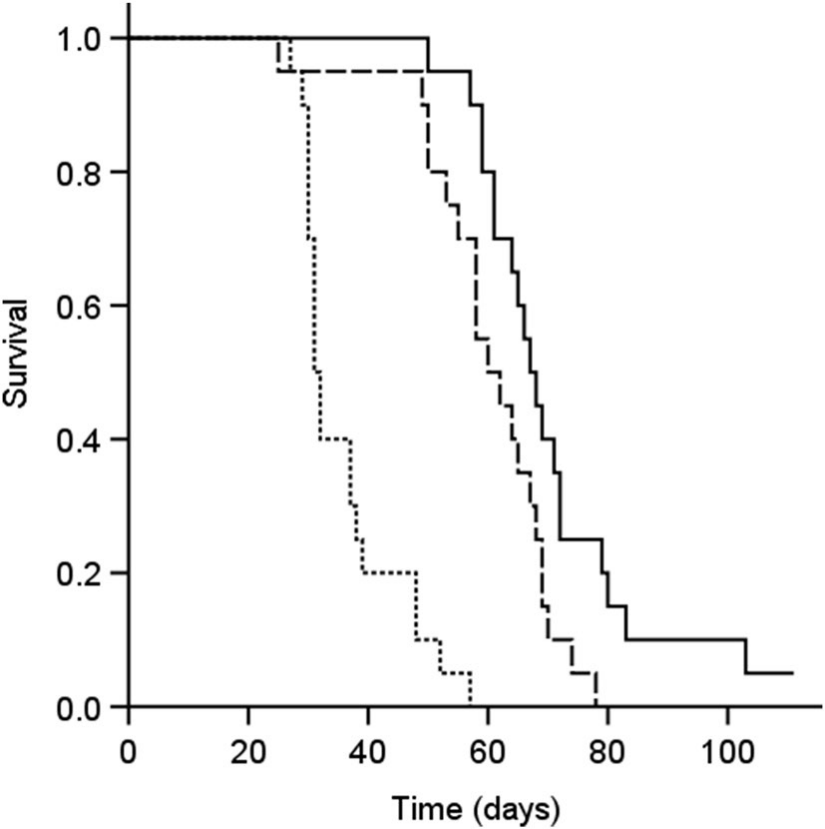
Survival in 60 rats with high-grade pseudomyxoma peritonei with signet ring cells, stratified according to treatment regimen: the cytoreductive surgery group, treated with cytoreductive surgery (CRS) without intraperitoneal chemotherapy (IPEC) (dotted line, n = 20), the normothermic group treated with cytoreductive surgery CRS and IPEC with mitomycin C (MMC) at 35 ºC (dashed line, n = 20), the hyperthermic group, CRS and IPEC with MMC at 41 ºC (solid line, n = 20)

Animals were generally sacrificed because of abdominal distention as a sign of intraperitoneal tumor recurrence, in two cases with concurrent jaundice. Five animals were sacrificed for other reasons: intestinal obstruction caused by adhesions (day 25, NG), jaundice because of massive tumor growth in the liver hilum (day 39, CRSG), intestinal obstruction caused by adhesions and respiratory failure because of large mediastinal tumor (day 66, HG), poor general condition and eye-infection, autopsy revealed abundant intraperitoneal tumor and no ascites (day 103, HG), end of the observation period without clinical sign of intraabdominal recurrence, at autopsy a negligible amount of intraperitoneal tumor and no ascites was found (day 110, HG). Figure 4 demonstrates that in animals successfully treated, survival was improved by administration of IPEC and regional hyperthermia. Median survival (95% confidence interval) was 31 days (30–32) in the CRSG, 60 days (51–69) in the NG and 67 days (63–71) in the HG (CRSG vs. NG, p < 0.001; CRSG vs. HG, p < 0.001; NG vs. HG, p = 0.015).

### Tumor distribution at autopsy

**Fig. 5:**
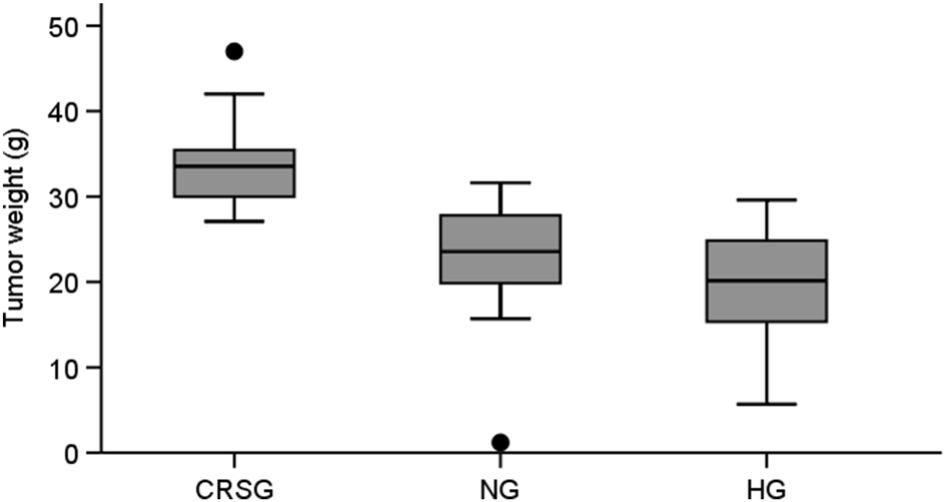
Tumor weight at autopsy illustrated by box plot of in 60 rats with high-grade pseudomyxoma peritonei with signet ring cells, stratified according to treatment regimen into the cytoreductive surgery group, treated with cytoreductive surgery (CRS) without intraperitoneal chemotherapy (IPEC) (CRSG, n = 20), the normothermic group treated with cytoreductive surgery CRS and IPEC with mitomycin C (MMC) at 35 ºC (NG, n = 20), the hyperthermic group, CRS and IPEC with MMC at 41 ºC (HG, n = 20)

At autopsy, abdominal distention was associated with hemorrhagic ascites and solid tumor characterized by denser consistency and more adherent growth to the peritoneal surfaces than at the time of surgery. Most animals had subcutaneous tumor implants in the laparotomy incision. No animals had metastasis to parenchymal organs (liver, lungs), but one animal in the HG developed a large mediastinal tumor. Generally, there was relatively less solid tumor tissue and more hemorrhagic ascites in the IPEC-treated animals compared to animals in the CRSG. In most animals, extensive peritoneal recurrence was observed, and tumor burden at this time was quantified by determining overall tumor weight. In detail, mean (SD) weight of solid tumor was 34 g (5) in the CRSG, 23 g (7) in the NG and 20 g (6) in the HG (CRSG vs. NG, p < 0.001; CRSG vs. HG, p < 0.001; NG vs. HG, p = 0.09) (Fig. 5). Mean (SD) weight of ascites was 31 g (14) in the CRSG, 65 g (18) in the NG and 54 g (24) in the HG (CRSG vs. NG, p < 0.001; CRSG vs. HG, p < 0.001; NG vs. HG, p = 0.15)

## Discussion

The combination of CRS and HIPEC is the only available curative treatment strategy for patients with PMP, but the relative importance of the individual components of HIPEC, intraperitoneal chemotherapy and regional hyperthermia, is still controversial. Animal studies with relevant tumor models where these components can be examined individually could therefore be useful. In a rare disease, such as PMP, randomized controlled trials are extremely challenging to perform. Given the large number of questions that need to be answered relating to issues such as HIPEC technique, carrier solution, choice of drug, drug dose, temperature and exposure time, there is a need for preclinical models to investigate some of these important details in patient management. The main findings of this study were that although no animals were cured, IPEC with MMC considerably improved survival and inhibited tumor growth compared to CRS alone, and addition of regional hyperthermia further improved the efficacy of IPEC.

The principle of HIPEC is that a high intraperitoneal drug concentration can be combined with limited systemic absorption. A key point is the concentration gradient between the perfusate and peritoneal tissues which causes absorption of the chemotherapeutic agent into peritoneal tissue and tumor according to Fick’s law of diffusion [20]. To obtain reproducible treatment conditions during IPEC, it seems logical to standardize drug concentration in the PPF rather than basing the drug dose on body surface area [21]. In this experimental series, the MMC dose was adjusted according to the volume needed to establish effective perfusion and almost identical MMC concentration was measured in all the animals. The chosen perfusion period, temperature and concentration were in line with conditions used in clinical HIPEC with MMC as proposed by van Ruth [8]. Thus, although animal experiments cannot fully capture all elements of the human disease and its treatment, in particular when complex therapeutic scenarios are investigated, by placing particular attention to study design assessment of individual therapy components is possible.

The model used in these experiments was established from a patient with high-grade PMP with signet ring cell differentiation and seems to relevantly mimic a challenging case of PMP with respect to growth pattern, histology, and surgical approach, in which a poor prognosis may be expected [13, 22]. Unfortunately, it was not possible to obtain complete removal of all tumor tissue (CC-0) in this model, mainly because of tumor deposits located in the liver hilum. Because of the soft tumor tissue in PMP, MMC is hypothesized to diffuse into small residual tumor nodules after CRS, suggesting that CC-1 cases will also benefit from administration of HIPEC [7]. This seems to also be the case in this experimental series, as survival was clearly improved by administration of IPEC with or without hyperthermia. The aggressive histology combined with CC-1 resection probably explains the inability of the procedure to cure the animals.

In the current model, a modest, but statistically significant increase in survival and a tendency towards reduction of tumor volume was observed when hyperthermia was added to CRS and IPEC. According to theory, hyperthermia may increase the efficacy of chemotherapy by modifying pharmacokinetics to increase tissue exposure and drug uptake, by potentiating drug cytotoxicity, and by impairing cellular repair mechanisms [23]. The rationale for adding regional hyperthermia to intraperitoneal chemotherapy was derived mainly from in vitro experiments in which increased cytotoxicity of MMC was demonstrated in gastrointestinal and breast cancer cell lines under hyperthermic conditions [11, 10]. Results from more recent clinical and preclinical studies are scant or contradictory. One clinical study with adjuvant HIPEC with MMC and cisplatin in gastric cancer demonstrated improved survival associated with addition of hyperthermia [24]. In contrast, the findings in an experimental study with a rat model of colorectal peritoneal metastases challenged the widely accepted role of regional hyperthermia in the treatment of patients with peritoneal surface malignancies, as hyperthermia did not improve survival after IPEC with MMC [25]. We previously demonstrated with the microdialysis technique that hyperthermia did not modify MMC pharmacokinetics in rats during IPEC, suggesting that survival benefit associated with hyperthermia should be attributed to pharmacodynamic effects [17].

The efficacy of adding HIPEC to CRS has been questioned by the results of the PRODIGE 7 trial, in which patients with peritoneal metastases from colorectal cancer underwent CRS and were randomized to HIPEC with oxaliplatin or no HIPEC [26]. There was no difference in overall survival between the groups and increased morbidity was observed in patients who received HIPEC. However, the results from this study are not automatically valid for other drugs and therapeutic settings, and therefore do not support abandonment of the HIPEC strategy in general [27]. Oxaliplatin and MMC are the most commonly used drugs in HIPEC in peritoneal metastasis of colorectal and appendiceal origin. Although both drugs are alkylating agents which damage tumor cell DNA, there are differences in mode of action on the molecular level [28, 29]. The drugs also differ in efficacy depending on tissue oxygenation, as MMC displays activity under hypoxic circumstances [30] while hypoxic tumor cells are resistant to oxaliplatin [31]. The pharmacokinetic profiles of the drugs are also different. Because of considerably faster absorption of oxaliplatin, with observed half-life in HIPEC of 30 min [32] versus 49 min for MMC [9], the chosen duration of the procedure is 30 min with oxaliplatin versus 90 min with MMC. Although both drugs are cell-cycle independent, this difference in drug exposure for peritoneal tumor could be critical for the anti-tumor effect. The results from this study provide experimental support for addition of MMC-based IPEC to CRS, and continued investigation of MMC as a HIPEC component is still warranted and relevant.

In conclusion, adequate imitation of patient treatment with CRS and HIPEC was achieved in an orthotopic tumor model of high-grade PMP with signet ring cell differentiation. Survival was improved and tumor growth was inhibited by CRS and IPEC with MMC compared to CRS alone, and outcome was further modestly improved by adding regional hyperthermia. In sum, the findings of this work support the continued addition of HIPEC with MMC for treatment of patients with PMP.

## Abbreviations

PMP: Pseudomyxoma peritonei
CRS: Cytoreductive surgery
HIPEC: Hyperthermic intraperitoneal chemotherapy
IPEC: Intraperitoneal chemotherapy
MMC: Mitomycin C
CRSG: Cytoreductive surgery group
NG: Normothermic group
HG: Hyperthermic group
PMCA: Peritoneal mucinous carcinomatosis
PCI: Peritoneal cancer index
PPF: Peritoneal perfusion fluid
SD: Standard deviation
AUC: Area under the curve
